# Multivariate empirical mode decomposition–based structural damage localization using limited sensors

**DOI:** 10.1177/10775463211006965

**Published:** 2021-03-31

**Authors:** Sandeep Sony, Ayan Sadhu

**Affiliations:** Department of Civil and Environmental Engineering, 120460 Western University, Canada

**Keywords:** Structural health monitoring, damage localization, multivariate empirical mode decomposition, damage index, limited sensors

## Abstract

In this article, multivariate empirical mode decomposition is proposed for damage localization in structures using limited measurements. Multivariate empirical mode decomposition is first used to decompose the acceleration responses into their mono-component modal responses. The major contributing modal responses are then used to evaluate the modal energy for the respective modes. A damage localization feature is proposed by calculating the percentage difference in the modal energies of damaged and undamaged structures, followed by the determination of the threshold value of the feature. The feature of the specific sensor location exceeding the threshold value is finally used to identify the location of structural damage. The proposed method is validated using a suite of numerical and full-scale studies. The validation is further explored using various limited measurement cases for evaluating the feasibility of using a fewer number of sensors to enable cost-effective structural health monitoring. The results show the capability of the proposed method in identifying as minimal as 2% change in global modal parameters of structures, outperforming the existing time–frequency methods to delineate such minor global damage.

## 1. Introduction

Structural health monitoring (SHM) has emerged as a foundational concept in the management and rehabilitation of critical civil infrastructure. The aging infrastructure, varying environmental effects, and other factors, such as increasing traffic conditions, have created a burden on maintaining the desired residual capacity of the structures. The encumbrance on civil structures because of such factors inherently induces damage in the structures, thereby compromising structural integrity and demanding timely maintenance. Damage identification and its localization (Burgos et al., 2020) are one of the critical steps toward efficient monitoring and maintenance of the structures. Through continuous monitoring, damages can be detected in the structures to prevent their major catastrophes at the later stage. In this article, a new and cost-effective damage localization technique is proposed to detect and isolate damage using a limited number of sensors.

Vibration-based SHM techniques (Barthorpe and Worden 2020; Cawley 2018; Erazo et al., 2019; Gatti 2019; Okayasu and Yamasaki 2019) offer viable options for tracking damages in the structures based on the measured data. Existing damage identification techniques involve analysis in time domain, frequency domain, and time–frequency (TF) domain (Sirca and Adeli 2012) along with various artificial intelligence techniques (Salehi and Burgueno 2018; Sony et al., 2020; Ying et al., 2012). In a structure, the damage is initiated because of changes in the structural or boundary condition of the system. As these properties change with time, the structure is mathematically modeled as a time-varying system (Chen et al., 2019; Zhou and Li 2017). Damage could be either discrete (because of a sudden shock) or progressive (because of a frequent accumulation of instantaneous damages) that can lead to a catastrophic failure. The time-domain methods only provide information about the time characteristics of the systems. Hence, they are prone to noise contamination and environmental factors, which offer challenges for large structures. On the other hand, time-series methods (Bernal et al., 2002; Johnson et al., 2004; Lei et al., 2003; Nair et al., 2006) require model order selection which is one of the limitations of these methods, especially for large-scale measurements with noise. Frequency-domain methods provide information about the frequency characteristics of the systems. Although frequency changes in the structure can be associated with the presence of damage, discrete changes in natural frequencies may not be sufficient for unique identification of the location of structural damage as a crack at two different locations may cause the same frequency change irrespective of its location (Salawu 1997). Time–frequency method (Neild et al., 2003) can identify the frequency components of the signal and apprehend their time-variant features.

Popular TF methods are short-time Fourier transform (STFT) (Daubechies 1990), Wigner–Ville distribution (WVD) (Andria et al., 1994), and empirical mode decomposition (EMD) (Barbosh et al., 2020; Huang et al., 1998). Short-time Fourier transform is used to increase the sparsity of the signals in TF domain, and the TF resolution of STFT is inversely proportional to window length. Increasing the window length improves the frequency resolution, whereas it decreases its frequency tracking capability. On the other hand, WVD has an unwanted cross-product disturbance along with the frequency bands, and there is a challenge to reconstruct the signal. Therefore, conventional TF analysis suffers from a trade-off between time and frequency resolution. Moreover, vibration data attribute highly nonlinear and nonstationary behavior due to various natural hazards such as wind and earthquake-induced excitation, where traditional TF analysis is not adequate. To address these issues, the nonlinear and nonstationary nature of vibration data was analyzed by Hilbert–Huang transform (HHT) (Huang et al., 1998), which is basis-free in nature. The HHT method can decompose any complex signal into a finite number of intrinsic mode functions (IMFs), which can be used to estimate modal parameters. A variant of EMD, multivariate EMD (MEMD) was recently explored as a modal identification tool using multi-sensor data (Barbosh et al., 2018; Sadhu 2015). Recently, Sony and Sadhu (2020) used a hybrid method combining MEMD and synchrosqueezing transform to study the behavior of time-varying structural systems. However, the important aspects of damage localization were not considered. In this article, a novel MEMD–based damage localization method is proposed that uses a limited number of sensors.

There have been continuous improvements in sensors and sensing technology as they constitute a significant portion of cost during SHM applications and contribute to the overall accuracy of condition assessment. Traditionally, wired sensors are used as a dense array distributed over the structure to acquire long-term SHM data. However, this approach is not a cost-effective and viable option for large-span bridges or tall buildings because of labor-intensive cable installation. The setbacks of wired sensors were envisioned to solve using smart wireless sensors (Abdulkarem et al., 2019; Lynch, 2007). Capabilities and local processing of wireless sensors in the decentralized framework were much later exploited by Spencer et al. (2004). Recently, other modern sensors such as cameras, robotic sensors, smartphones, and drones have been used for SHM through the processing of images and videos (Feng and Feng 2018; Sony et al., 2019). However, such SHM approach is cost-effective only if the hidden structural information and damage characteristics are accurately assessed from the limited measured data.

An optimal number of limited sensors (Li et al., 2014) that can provide the same information as the large array of sensors will reduce the cost of SHM. There has been considerable research on system identification by limited sensors using two popular methods: non-sparse and sparse techniques. Non-sparse methods have gained interest in the operational modal analysis as a nonparametric alternative to the structural identification from output-only measurements (Hazra et al., 2011; Sadhu et al., 2017, 2019). A parallel factor decomposition along with Bayesian model updating was used for underdetermined modal identification (Abazarsa et al., 2013). In addition, Guo and Kareem (2016) proposed spatial TF distribution to handle nonstationary response. However, the proposed method highly depends on the quality of the selection of a single auto-spectral component, and its poor selection could lead to inaccurate results.

Unlike non-sparse methods, sparse techniques use TF methods for sparse representation of signals. Yang and Nagarajaiah (2013) used sparse component analysis (SCA) along with L1-minimization to improve underdetermined modal identification. The study by Yu et al. (2014) explored SCA-based methods in the TF domain to estimate time-varying modal parameters and validated the proposed method under thermal effects. Amini and Hedayati (2016) used STFT and SL0 algorithm to perform underdetermined system identification using earthquake and ambient excitation and showed the robustness under noise to separate closely spaced modes. Moreover, in Yao et al. (2018), the authors explored SCA for modal identification using limited sensors and validated the method on both stationary and nonstationary response of structures. Castiglione et al. (2018) proposed frequency banding for largely underdetermined scenarios by decomposing a large underdetermined problem into several overdetermined problems. The method operates directly in the frequency domain and analyzes the cross-spectral matrix of the data. However, user intervention during frequency banding and manual selection of estimated modes is one of the limitations of the proposed method. Yi et al. (2018) proposed a novel SCA method for estimating the number of active modes using statistical properties of normalized single source point vector. Although the above sparse and non-sparse system identification methods show a wide variety of applications in modal identification using limited sensors, damage localization has not given its due attention under limited sensor measurements (Gomes et al., 2018).

Amini and Karami (2012) recently used the system Markov parameters for damage detection. The authors evaluated the effect of noise, number, and location of the sensors. The study was based on finite element study and predefined damage locations and did not identify the unknown damage location. Bagheri et al. (2016) used an optimization algorithm to localize and quantify damage in seismically excited structures using a limited number of sensors. A competitive optimization algorithm combined with moment generating function was used as a damage indicator. The limitation of the study lies in using the moment of the segment as a damage indicator while identifying the instance of the damage. Recently, Karami-Mohammadi et al. (2020) used mode shapes coupled with wavelet transform for damage identification while using a limited number of sensors. The optimal spatial location of the sensors was achieved using the minimization of the non-diagonal entries in the modal assurance criterion matrix. The study is dependent explicitly on mode shapes, and ambiguity arises on interpreting the damage location with respect to different damage scenarios. Moreover, there is a significant challenge in accurate identification of mode shapes using real data with measurement noise.

In this article, MEMD–based damage localization is proposed by taking advantage of the modal energies of individual modes extracted from the measured data. Because of the capability of handling a limited number of sensors, MEMD is further explored to compare the performance of the damage localization using a suite of limited sensors. The article is organized as follows. Section 1 provides a brief introduction to this topic, illustrating the existing literature and the gap areas. Section 2 presents the proposed methodology and the corresponding theoretical background. Section 3 shows the numerical studies using a 10-DOF model subjected to a wide extent of damage and measurements obtained from a suite of limited sensors. A full-scale study comprising of a real bridge with two different damage scenarios is presented in Section 4, followed by key conclusions in Section 5.

## 2. Background of MEMD

A brief background of MEMD method is presented first before discussing the details of the proposed algorithm. Multivariate EMD is a variant of EMD (Huang et al., 1998) and EMD works only for a single measurement. The basic idea of EMD is that any complicated dataset can be decomposed into a finite number of “intrinsic mode functions (IMFs).” This decomposition method is adaptive and, therefore, free of any basis function. Because the decomposition is based on the local characteristics of the data, it is applicable to nonlinear and nonstationary processes. In the presence of multiple sensors, EMD faces several challenges (Sadhu, 2015) such as (a) there is no guarantee that the extraction of IMFs from different channels of measurements will match, either in number or in their frequency contents, due to their sole dependencies on a single measurement, and (b) the joint information between multichannel measurements is not considered because signals from multiple sensors are treated individually. For multichannel measurements, a multivariate extension (i.e., MEMD) is proposed by Rehman and Mandic (2010). Because of the geometric projection and averaging tasks, MEMD is free of any linear algebraic operations and allows solving of underdetermined system identification using limited sensors.

Vibration-based monitoring of real-life structures requires use of multiple sensors and structural modes that are closely spaced. An array of sensors for a large-scale structure is expensive and takes a considerable portion of budget of the condition assessment. In this article, MEMD is used to reduce the number of sensors by decomposing the signal into its IMFs. A suitable set of direction vectors are sampled on unit hyperspheres (*n*-spheres) based on both uniform angular sampling methods and quasi-Monte Carlo–based low-discrepancy sequences. To estimate all multidimensional envelopes, the multiple signals are projected onto the chosen direction vectors and the average of all envelopes is considered as a local mean of multiple signals. The algorithm for MEMD (Rehman and Mandic 2010) is presented in Algorithm 1.

**Algorithm 1:** MEMD

**Input:** A signal *y*(*t*)

**Result:** Intrinsic mode functions (IMFs)

Initialization

**for** *y*(*t*) = signal **do**

 1. Evaluate the direction vector, *D* and its projections along *k* − th direction;

  2. Determine the *k*-th projection, *p*^*k*^(*t*) of the input signal *y*(*t*) over the *k*-th direction vector, *Y*^*k*^, for all *k* (*k*=1, 2,….., *L*, where *L* is the number of direction vectors *D*).

  3. Estimate the corresponding time $t_{i}^{k}$ of maximum *p*^*k*^(*t*) for all *k*.

  4. Interpolate $\left[t_{i}^{k},\;y\left(t_{i}^{k}\right)\right]$ to extract multi-dimensional envelopes, *ϵ*^*k*^(*t*).

  5. Determine the mean of envelope $\bar{W}\left(t\right)$ as,

(1) $$\bar{W}\left(t\right)=\frac{1}{L}\sum_{k=1}^{L}\epsilon^{k}\left(t\right)$$

  6. Estimate the residual *R*(*t*) using $R\left(t\right)=y\left(t\right)-\bar{W}\left(t\right)$ . If *R*(*t*) satisfies the stopping criteria of multivariate IMF, repeat the above steps to [*y*(*t*) − *R*(*t*)] until the next IMF is diminished. Otherwise, repeat it to *R*(*t*).

End

## 3. Proposed algorithm

Consider a linear dynamical system with *n* degree-of-freedom (DOF) subjected to a broadband excitation X(*t*), the equation of motion is expressed as

(2) $$M\overset{..}{y}\left(t\right)+C\dot{y}\left(t\right)+Ky\left(t\right)=\left(t\right)$$

where *y*(*t*) is the displacement vector. Using the classical modal superposition theorem, the solution to equation (2) for those of broadband **X**(*t*) can be written in terms of an expansion of vibration modes

(3) y=Φν

where **y** and ***ν*** are the response and modal response matrix, respectively. Φ_*m*×*n*_ is the modal transformation matrix. *n* and *m* are the number of modal responses and measurements, respectively. The measurement at the *i*-th DOF (*i* = 1, 2, …, *m*) can be expressed as

(4) $$y_{i}\left(t\right)=\sum_{j=1}^{n}\phi_{ij}\nu_{j}\left(t\right)$$

where *ν*_*j*_ is the *j*-th modal response and *ϕ*_*ij*_ represents the mode shape ordinate of *i*-th DOF and *j*-th mode. Because MEMD results in IMFs containing mono-component modal responses, each signal of **y**(*t*) can be expressed in terms of its IMFs

(5) $$y_{i}\left(t\right)=\sum_{j=1}^{n}I_{ij}\left(t\right)$$

where *I*_*ij*_ is the *j*-th IMF of *y*_*i*_(*t*). Comparing the equality of equations (4) and (5), we get

(6) $$I_{ij}\left(t\right)=\phi_{ij}\nu_{j}\left(t\right)\;i=1,2,\ldots ,\;m$$

Each IMF *I*_*ij*_ is then analyzed to obtain Fourier spectrum (*E*_*ij*_(*f*)), as shown in equation (7)

(7) $$E_{ij}\left(f\right)=\int_{-\infty }^{\infty }I_{ij}\left(t\right)e^{-kft}dt$$

If *E*_*ij*_(*f*) is the Fourier transform of *I*_*ij*_(*t*), then |*E*_*ij*_(*τ*)|^2^ can be interpreted as energy density at the frequency *τ*, which means that the total energy contained in a small frequency interval [*τ* − *ϵ*, *τ* + *ϵ*] around *τ* is approximately given by 2*ϵ*|*E*_*ij*_(*τ*)|^2^. However, as per mathematical axiom, if |*x*| > |*y*|, then, for every *x*≠0, *y*≠0, and *x*, *y* > 0, *x*^2^ > *y*^2^ is true, and thus, the peak Fourier amplitude can be interpreted as modal energy. The damage localization feature is then extracted using percentage change in peak amplitude of Fourier spectrum of modal responses as shown in equation (8)

(8) $$\Delta E_{ij}=\left|\frac{\left(E_{ij}\left(f_{D}\right)\right)-\left(E_{ij}\left(f_{U}\right)\right)}{E_{ij}\left(f_{U}\right)}\right|\times 100$$

where Δ*E*_*ij*_ represents absolute percentage change in modal energy of the corresponding damaged (*f*_*D*_) and undamaged (*f*_*U*_) modal frequencies. Then, Δ*E*_*ai*_ is calculated as the average of Δ*E*_*ij*_ for all selected IMFs of *i*-th sensor

(9) $$\Delta E_{ai}=\frac{\sum_{j=1}^{n}\Delta E_{ij}}{n}$$

Finally, the threshold $\bar{\Delta E}$ is proposed by taking mean value of Δ*E*_*ai*_ for all the selected sensor locations

(10) $$\bar{\Delta E}=\frac{\sum_{i=1}^{m}E_{ai}}{m}$$

The proposed algorithm is applied by first decomposing the multi-sensor data into their IMFs, and then, their Fourier spectra are used to identify Δ*E*_*ij*_ of the corresponding frequencies and are used as damage localization feature. The damage is deemed present for any sensor location, if Δ*E*_*ai*_ is higher than $\bar{\Delta E}$ . The flowchart of the proposed algorithm is presented in Algorithm 2.

**Algorithm 2:** Proposed Algorithm

**Input:** A signal *y*(*t*)

**Result:** Damage localization feature, $\bar{\Delta E}$

Initialization,

**for** *y*(*t*) = signal **do**

  1. Determine direction vector, *D* and its projections along *k*-th direction;

  2. Find the maxima of the projected signal *p*^*k*^(*t*) for all *k*;

  3. Interpolate $\left[t_{i}^{k},\;y\left(t_{i}^{k}\right)\right]$ to extract the multi-dimentional envelopes, *ϵ*^*k*^(*t*) and IMF, *I*_*ij*_ obtained from MEMD.

  **for** IMFs, (*I*_*ij*_) **do**.

   (a) Calculate *E*_*ij*_(*f*)

   (b) Calculate percentage change in modal energy, Δ*E*_*ij*_ of corresponding damaged $\left(E_{ij}\left(f_{D}\right)\right)$ and undamaged (*E*_*ij*_(*f*_*U*_)) modal frequencies.

   (c) Find Δ*E*_*ai*_.

   (d) Calculate threshold, $\bar{\Delta E}$ by taking mean of the corresponding Δ*E*_*ai*_ for all measurements.

  End.

End.

## 4. Numerical studies

A 10-DOF model is used to illustrate the performance of the proposed method. The model is subjected to a broadband earthquake (i.e., Imperial Valley earthquake) with a peak ground acceleration (PGA) of 0.1 g to study the damage localization using the earthquake-induced measurements. Considering the fact that the first floor columns are primarily damaged during the earthquakes, three damage scenarios are used to evaluate the performance of the proposed method under varying level of damage in the first floor. For example, C1 indicates undamaged model with first five natural frequencies as 0.78, 1.8, 2.83, 3.88, and 4.96 Hz, respectively, whereas C2 and C3 represent the 10-DOF model with 20% and 40% damage in the column of the first floor, respectively. Table 1 illustrates the corresponding natural frequencies of the 10-DOF model under C2 and C3, and the associated percentage changes in the natural frequencies with respect to C1. It can be observed that the damage cases are chosen such a way that the overall average reduction in modal frequencies are less than 5% in C2 and C3 (i.e., 1.9% and 4.5%, respectively), where the traditional TF methods (Sony and Sadhu 2020) were unable to delineate such minor change in the modal frequencies. The models are subjected to above earthquake and the earthquake-induced responses are analyzed to identify the location of the simulated damage.

**Table 1.** The first five modal frequencies of the 10-degree-of-freedom model for various damage cases and their percentage reduction with respect to C1.

	Modal frequency (Hz)				
	*f*_1_ (% change)	*f*_2_ (% change)	*f*_3_ (% change)	*f*_4_ (% change)	*f*_5_ (% change)
C2	0.76 (2.6)	1.76 (2.2)	2.78 (1.8)	3.82 (1.5)	4.90 (1.2)
C3	0.73 (6.4)	1.70 (5.9)	2.71 (4.2)	3.75 (3.4)	4.83 (2.6)

**Figure 1.**
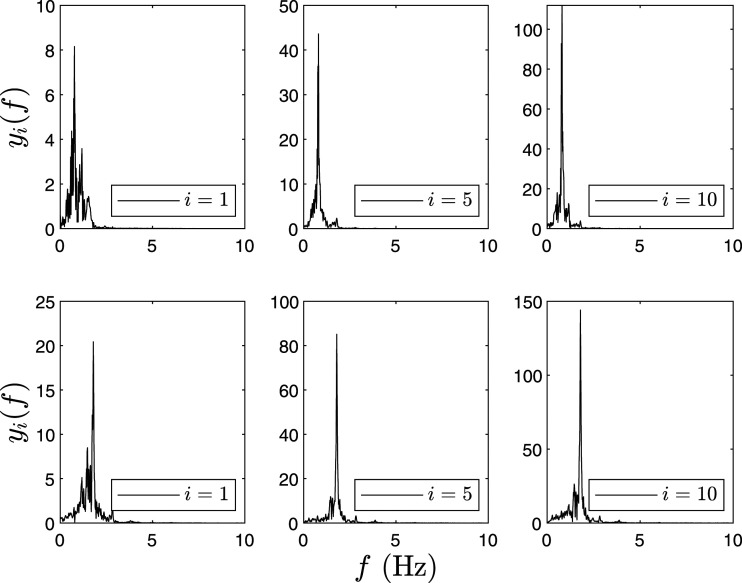
Fourier spectra of IMF-1 and IMF-2 of the 10-DOF model for C1. Note: DOF: degree-of-freedom; IMF: intrinsic mode functions.

**Figure 2.**
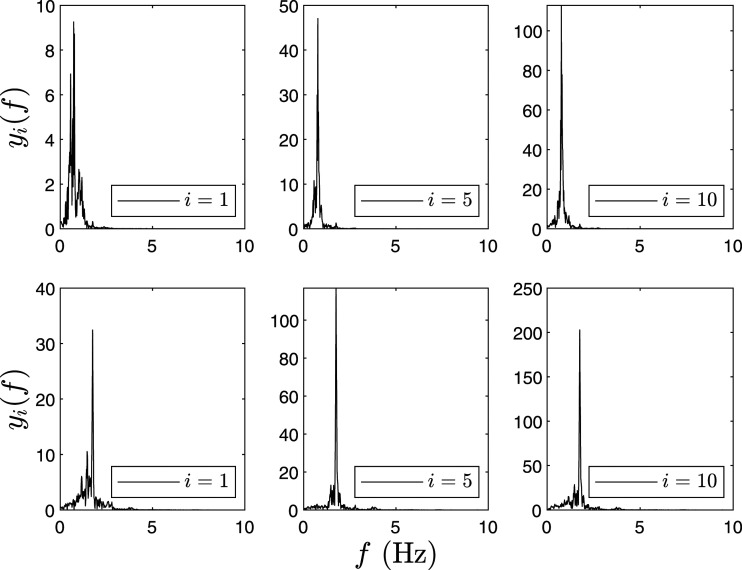
Fourier spectra of IMF-1 and IMF-2 of the 10-DOF model for C2. Note: DOF: degree-of-freedom; IMF: intrinsic mode functions.

**Figure 3.**
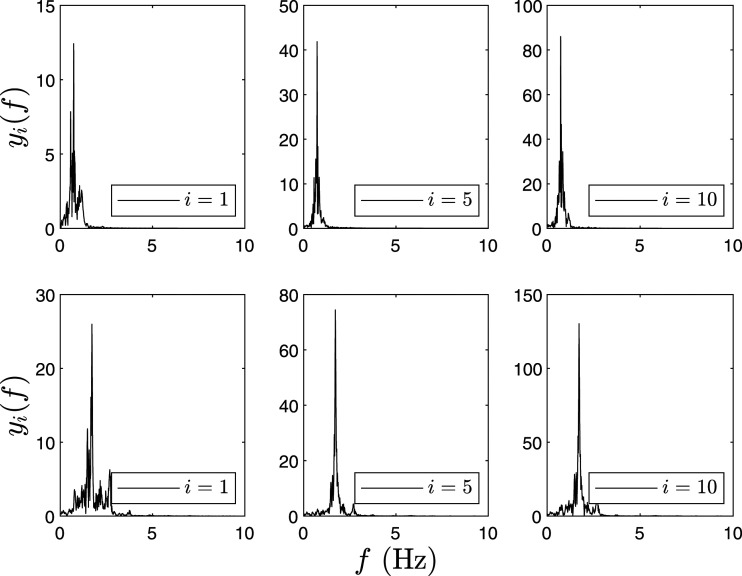
Fourier spectra of IMF-1 and IMF-2 of the 10-DOF model for C3. Note: DOF: degree-of-freedom; IMF: intrinsic mode functions.

The 10-DOF model is excited using Imperial Valley earthquake and damage is induced at the first floor (*i* = 1) by reducing the stiffness, and the proposed algorithm is evaluated to localize damage using a combination of all sensors. MEMD is implemented to decompose the simulated responses into their IMFs. The first two modes are illustrated to show the change in the natural frequencies and Δ*E*_*ai*_ is used as a damage indicator. The values of Δ*E*_*ij*_ for various floors of *I*_*i*1_ (IMF-1) and *I*_*i*2_ (IMF-2) are tabulated in Tables 2 and 3, respectively. For example, Table 2 provides the peak Fourier amplitude of first floor response as 8.2 unit for C1, as shown in Figure 1(a). In Figure 1–3, the first row shows IMF-1 and second row shows IMF-2, respectively. The corresponding modal energies for C1, C2, and C3 are shown in Figures 1–3, respectively.

**Table 2.** Modal energies (*E*_*ij*_(*f*)) of IMF-1 and IMF-2 of the 10-DOF model subjected to Imperial Valley earthquake.

	C1 (Figure 1)		C2 (Figure 2)		C3 (Figure 3)	
Floor (*i*)	IMF-1	IMF-2	IMF-1	IMF-2	IMF-1	IMF-2
1	8.2	20.4	8.7	32.4	12.4	26
5	43.6	85.1	47.1	116.9	41.9	74.4
10	112	144.1	112.8	202.8	86	130.2

Note: DOF: degree-of-freedom; IMF: intrinsic mode functions.

**Table 3.** Δ*E*_*ai*_ for the various floors.

	C2–C1		C3–C1		Δ*E*_*ai*_	
Floor (*i*)	Δ*E*_*i*1_	Δ*E*_*i*2_	Δ*E*_*i*1_	Δ*E*_*i*2_	C2 (Figure 4(a))	C3 (Figure 5(a))
1	6.1	58.8	51.2	27.5	32.5	39.3
5	8.0	37.4	3.9	12.6	22.7	8.2
10	0.7	40.7	23.2	9.6	20.7	16.4

**Figure 4.**
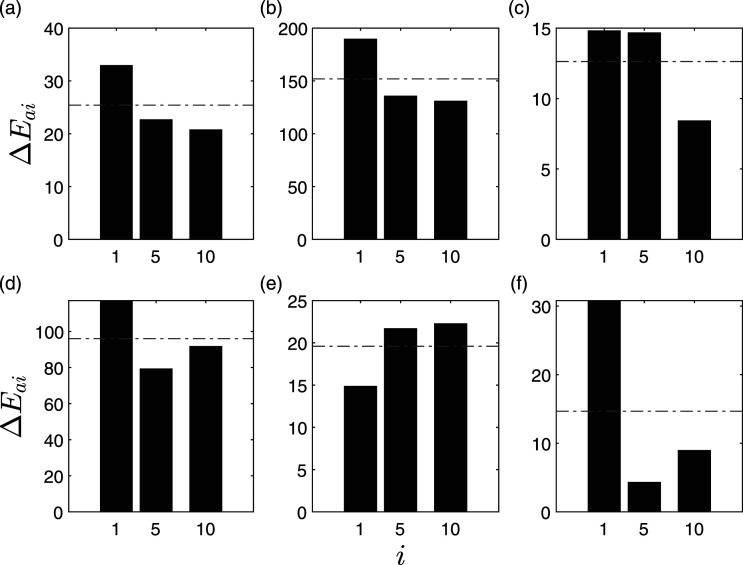
Δ*E*_*ai*_ of intrinsic mode functions for C2 subjected to Imperial Valley EQ using (a) 10 sensors, (b) 9 sensors, (c) 8 sensors, (d) 7 sensors, (e) 6 sensors, and (f) 5 sensors.

To localize the damage, equation (8) is used to calculate Δ*E*_*ij*_, followed by evaluating Δ*E*_*ai*_ using equation (8) for all selected IMFs of a sensor. Finally, the average of Δ*E*_*ai*_ for all sensors is evaluated using equation (9) and is used as a damage localization feature, as presented in Table 3. The percentage change between C1 and C2 is 6.1% for IMF-1, similarly, for C1 and C3, its 51.2%. The damage localization feature is then evaluated by taking mean of Δ*E*_*ij*_ of all IMFs. For example, C2 yields Δ*E*_*ai*_ as 32.5 unit, which is equal to average of corresponding values of IMF-1 and IMF-2. Δ*E*_*ai*_ values for various floors are shown in Figure 4(a). For example, the first floor has a value of 32.5 unit, the fifth floor has a value of 22.7 unit, and 10th floor has a value of 20.7 units. In this sub-figure, $\bar{\Delta E}$ is calculated as 25.3 unit, which is shown in the dotted line and it can be clearly seen that the proposed damage index exceeds $\bar{\Delta E}$ at *i* = 1, indicating accurate damage localization at the first floor.

**Figure 5.**
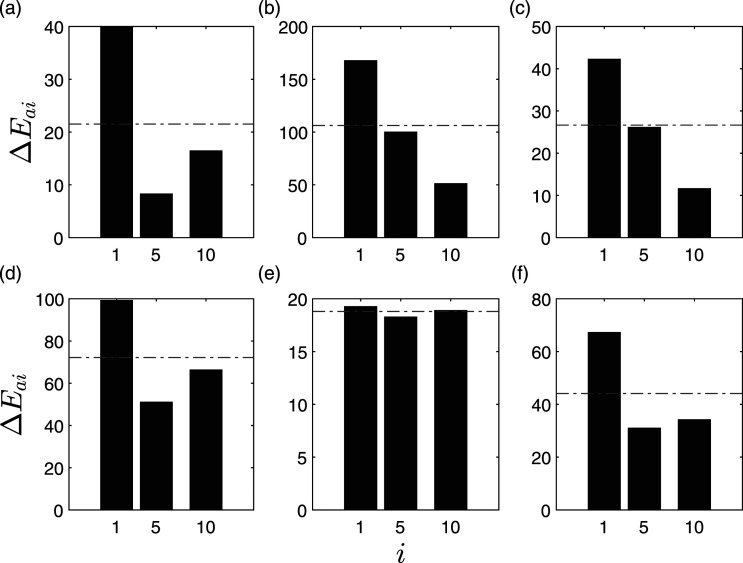
Δ*E*_*ai*_ of intrinsic mode functions for C3 subjected to Imperial Valley EQ using (a) 10 sensors, (b) 9 sensors, (c) 8 sensors, (d) 7 sensors, (e) 6 sensors, and (f) 5 sensors.

The study is further enunciated by considering a limited number of sensors, whereas keeping sensor locations 1, 5, and 10 intact for all the cases to have consistent reference sensors for the comparison purposes. For example, Figure 4 shows the identification results of C2 for a limited sensor cases using 10, 9, 7, and five sensors, respectively. The damage location can be clearly identified in floor 1; however, in case of eight and six sensors, the proposed method could not classify accurately. In case of C3, all the sensors classify the damaged floor accurately, as shown in Figure 5. Therefore, the sensitivity of the proposed method improves with the severity of the damage.

The classification or damage localization accuracy under various number of sensor locations and their combinations are summarized in Table 4, where 0 and 1 indicate index for misclassification and accurate classification, respectively. It can be concluded that the proposed method is sensitive to the severity of damage, the higher the severity, the higher is the accuracy for damage localization. However, the proposed method has nearly 92% accuracy across all limited sensor cases in C2 and C3 subjected to the example earthquake, indicating its efficacy to accurately identify less than 5% global change in the modal frequencies.

**Table 4.** Damage localization accuracy of the 10-degree-of-freedom model using the limited number of sensors.

No. of sensors	10	9	8	7	6	5
C2	1	1	1	1	0	1
C3	1	1	1	1	1	1

## 5. Full-scale study

A benchmark study on Z24 bridge is used to evaluate the performance of the proposed method. The bridge is located in Canton Bern, Switzerland, connecting Koppigen and Utzenstorf. The bridge is a highway overpass linking Bern and Zurich and it is a prestressed bridge, with three spans, two lanes, and 60 m overall length (Maeck and Roeck De (2003);

Teughels and Roeck (2004)). The bridge was demolished at the end of 1998 because a new railway adjacent to the highway required a new bridge with a larger side span. The bridge was excited by two shakers, one at the mid-span of the bridge and another at the side span. Because of the size of the bridge, response was measured in nine setups with each setup containing up to 15 sensors each. In all setups, three accelerometers and the two force sensors were common. The data were sampled at 100 Hz, and a total of 65,536 samples were acquired.

**Figure 6.**
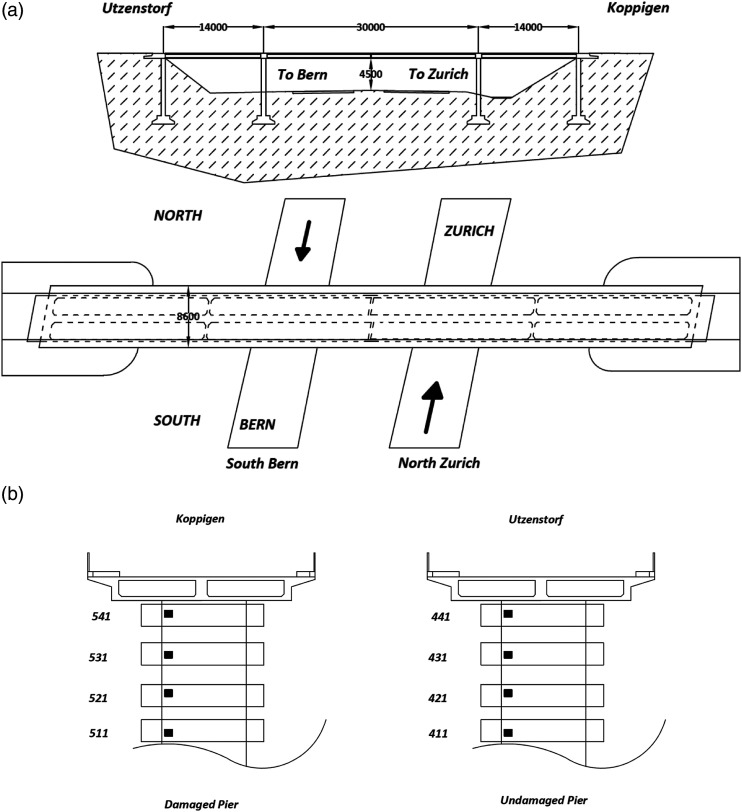
(a) Schematic of the Z24 bridge and (b) schematic of damaged (Koppigen) and undamaged pier (Utzenstorf).

The study has multiple damage cases; however, for the purpose of the damage localization, only three cases are considered with a total number of eight sensors, as shown in Figure 6. The pier settlement case was used to localize the damage and differentiate the damaged pier from the undamaged pier. The settlement was simulated by cutting the Koppigen pier and removing about 0.4 m of concrete. Lowering and lifting was applied by six hydraulic jacks. During the tests, the pier rested on steel sections with similar stiffness as the uncut concrete section. The lowering of the pier was carried out by supporting the structure with scaffolding. The pier was cut to support its dead weight, test equipment, and the impact of a vehicle with and without normal force. The base plates were located and connected using shear connectors. One of the piers (Koppigen) among two piers, as shown in Figure 6, was damaged by lowering the piers. In this study, first, an undamaged case was used as a baseline and then a pier settlement of 20 mm, 40 mm, and 95 mm are used as the damaged cases. Schematic and sensors of the undamaged and damaged piers used in this study are shown in Figure 6. These data were made publicly available by researchers at the Katholieke Universiteit Leuven and is available at: https://bwk.kuleuven.be/bwm/z24.

**Figure 7.**
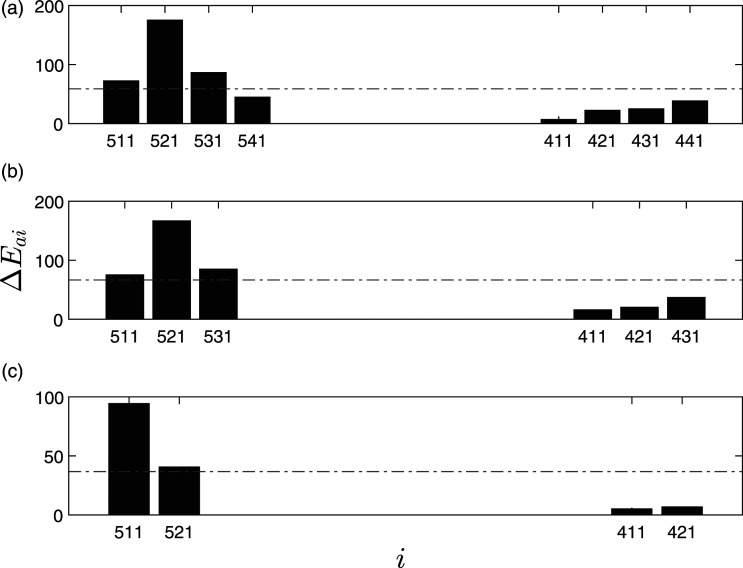
Δ*E*_*ai*_ for 20 mm lowering of pier under various sensor cases using (a) 8 sensors, (b) 6 sensors, and (c) 4 sensors.

**Figure 8.**
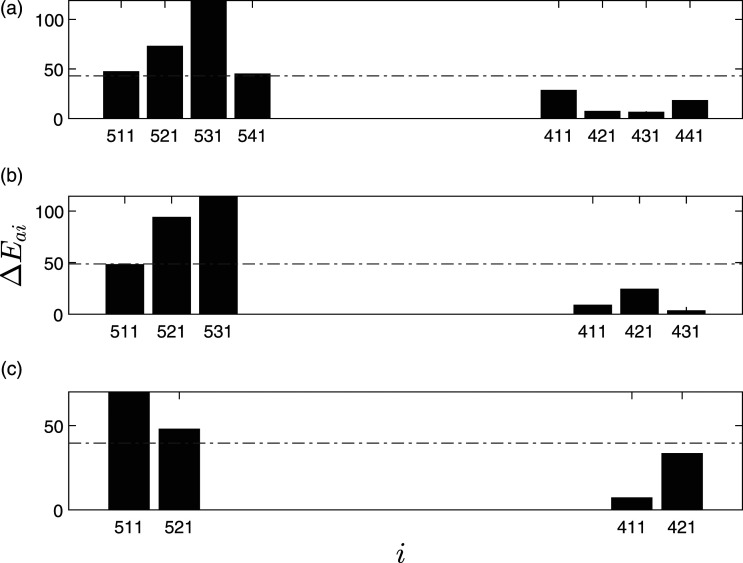
Δ*E*_*ai*_ for 40 mm lowering of pier under various sensor cases using (a) 8 sensors, (b) 6 sensors, and (c) 4 sensors.

**Figure 9.**
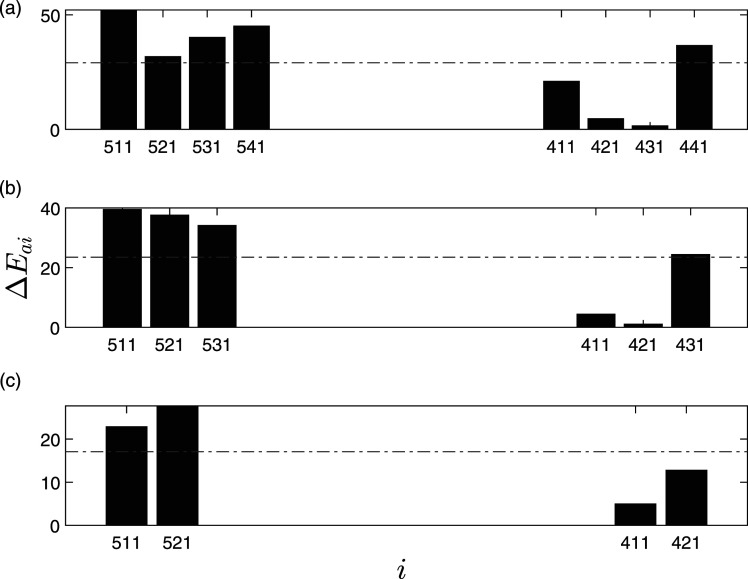
Δ*E*_*ai*_ for 95 mm lowering of various sensor cases using (a) 8 sensors, (b) 6 sensors, and (c) 4 sensors.

The damaged pier is localized using the proposed algorithm. Various cases of limited sensors are used to illustrate the performance of the proposed algorithm. There are four sensors each on both of the piers (a total of eight sensors), and for limited sensor case, the number of sensors was reduced to six and four, respectively. $\bar{\Delta E}$ of each sensor is used to present the damage localization feature for various cases. Three different damage localization scenarios of pier settlement are considered, namely 20 mm, 40 mm, and 95 mm of settlement to include the severity of damage in the analysis. As shown in Figure 7, for 20 mm pier settlement, it can be observed that Δ*E*_*ai*_ of the damaged pier is consistently higher than the $\bar{\Delta E}$ in all limited sensor cases. Similar analysis is conducted for 40 mm and 95 mm lowering of pier as shown in Figures 8 and 9, respectively. The results show that the proposed method is able to detect the damage with various severities as well as identify the location of damage using a suite of limited sensor cases across various levels of damage.

**Figure 10.**
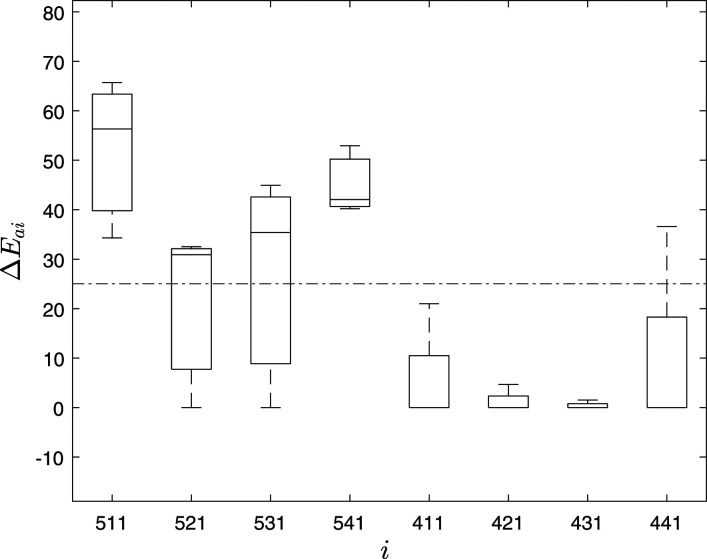
Δ*E*_*ai*_ of intrinsic mode functions of the Z24 bridge using 8 sensors.

**Figure 11.**
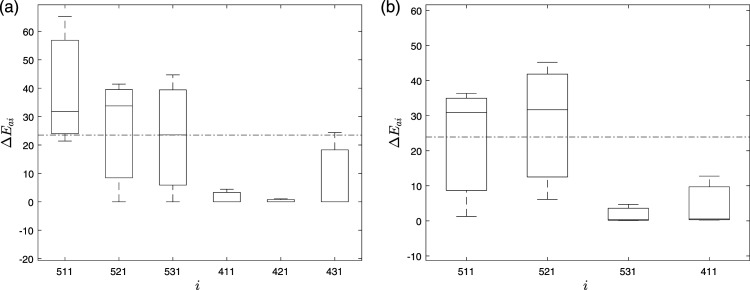
Δ*E*_*ai*_ of intrinsic mode functions of the Z24 bridge using (a) 6 sensors and (b) 4 sensors.

The variability for 95 mm pier settlement in Δ*E*_*ai*_ for each mode of every sensor is presented as box plots and for various limited sensors damage cases, as shown in Figures 10 and 11(a) and (b), respectively. It can be observed that along with mean of Δ*E*_*ai*_ for each sensor, the variability in the modal Δ*E*_*ai*_ for the damaged cases is larger than $\bar{\Delta E}$ , indicating clear delineation of the damage location at the damaged pier. It can be observed that high variation is in the sensors from the damaged pier (i.e., 511, 521, 531, and 541) and low variation in the undamaged pier (i.e., 411, 421, 431, and 441). Moreover, the damage index of the damaged pier is always higher than the threshold value irrespective of the number of limited sensors, indicating the efficacy of the proposed method to identify the damage location.

## 6. Conclusions

In this article, a novel damage localization method is proposed using a damage index obtained from MEMD. Multivariate EMD is used to decompose multi-sensor data into their mono-components. The mono-component modal responses are further used to evaluate the modal energy to derive the damage localization feature using limited sensors. The threshold for the proposed damage index is adaptive and automated in nature and free of any user discretion or any predefined value, which is one of the merits of the proposed method. To the best of the authors’ knowledge, the capabilities of MEMD in performing damage localization using limited sensors have not been undertaken in the literature. The proposed method is demonstrated using a suite of numerical studies and the benchmark data of the Z24 bridge. It is concluded that the proposed method works well on all the studies and is effective in localizing the damage. The limited measurement aspect of damage localization is explored by selecting a fewer number of sensors and it is shown that with limited measurements, the proposed method is as effective as total number of measurements equals to the DOF of the model. The results show the capability of the proposed method in identifying as minimal as 2% change in global modal parameters of structures, outperforming the existing TF methods to delineate the minor global damage.
